# Patient-Reported Symptom Recovery After Upper Gastrointestinal Cancer Surgery: A Prospective Study Using the MDASI-UGI-Surg

**DOI:** 10.1245/s10434-026-19282-0

**Published:** 2026-02-19

**Authors:** Koichi Tomita, Paula M. Smith, Maho Takayama, Cong Pan, Shu-En Shen, Xuemei Wang, Xin Shelley Wang, Loretta A. Williams, Elsa M. Arvide, Connie To, Ravi Rajaram, Kyle G. Mitchell, David C. Rice, Wayne L. Hofstetter, Mara B. Antonoff, Reza J. Mehran, Ara J. Vaporciyan, Garrett L. Walsh, Jessica E. Maxwell, Rebecca A. Snyder, Michael P. Kim, Ching-Wei D. Tzeng, Paul F. Mansfield, Stephen G. Swisher, Jeffrey E. Lee, Brian D. Badgwell, Matthew H. G. Katz, Naruhiko Ikoma

**Affiliations:** 1 Department of Surgical Oncology, The University of Texas MD Anderson Cancer Center, Houston, TX USA; 2 Department of Symptom Research, The University of Texas MD Anderson Cancer Center, Houston, TX USA; 3 Department of Biostatistics, The University of Texas MD Anderson Cancer Center, Houston, TX USA; 4 Department of Thoracic and Cardiovascular Surgery, The University of Texas MD Anderson Cancer Center, Houston, TX USA

**Keywords:** Upper Gastrointestinal (UGI) Cancer Surgery, Patient-Reported Outcomes (PROs), Postoperative Symptom Burden, Cumulative Recovery Rate, Prospective Cohort Study, MD Anderson Symptom Inventory (MDASI)

## Abstract

**Background:**

Understanding patients’ symptom recovery after upper gastrointestinal (UGI) cancer surgery is essential for patient-centered care, yet detailed longitudinal patient-reported outcome data remain limited. We conducted a prospective study using the novel MD Anderson Symptom Inventory for UGI Surgery (MDASI-UGI-Surg) tool.

**Methods:**

Patients undergoing esophageal (*n* = 42), gastric (*n* = 27), or pancreatic (*n* = 74) cancer surgery from February to September 2024 were enrolled. The MDASI-UGI-Surg comprises 22 symptoms and 6 interference items. The five most severe symptoms and three most severe interference items on postoperative day (POD) 3 were identified. Recovery was defined as achieving mild severity for both symptom and interference composites. Multivariable analysis was performed to identify factors associated with recovery at postoperative month (POM) 1.

**Results:**

Most symptoms peaked at POD3. The five most severe symptoms were pain, fatigue, sleep disturbance, drowsiness, and dry mouth; the top three interference items were general activity, working, and enjoyment of life. Symptom recovery followed three phases: an acute improvement phase (POD3–14), a plateau phase (POD14–POM1), and a persistent recovery phase extending to POM6. Symptom profiles were similar across organ groups, and fatigue remained prolonged. Cumulative recovery rates were 64.8% at POM1, 78.9% at POM3, and 90.8% at POM6. Postoperative complications and readmissions were associated with delayed recovery at POM1, and the type of surgery predicted recovery in multivariable models.

**Conclusions:**

This study provides a detailed characterization of symptom recovery after UGI cancer surgery. These findings support improved preoperative counseling and postoperative care planning.

**Supplementary Information:**

The online version contains supplementary material available at 10.1245/s10434-026-19282-0.

“What will my recovery after surgery be like?” is one of the most frequent questions patients ask while preparing for life-altering cancer operations. Yet, surgeons often struggle to provide meaningful, data-driven answers. Offering overly optimistic expectations can lead to disappointment and erosion of trust and the patient–surgeon relationship, whereas excessively pessimistic messaging may cause undue anxiety and emotional distress before surgery.^1^ The treatment of upper gastrointestinal (UGI) cancers has become increasingly complex with advances in surgical techniques and multidisciplinary approaches, including neoadjuvant treatments and minimally invasive surgery.^2^ These evolving practices make accurate estimation of postoperative recovery even more challenging.

Traditional studies have relied on objective metrics, such as hospital stay length, complication rates, and return to adjuvant therapy, as surrogate indicators of recovery.^3–5^ However, these measures fail to capture the nuanced and subjective aspects of recovery that matter most to patients.^6,7^ Patients’ perceptions of recovery often diverge from those of surgeons, leading to miscommunication and unmet expectations. To close this gap, a patient-centered understanding of recovery is essential,^8^ and patient-reported outcomes (PROs) offer a validated method to assess health-related quality of life from the patient’s perspective.^9,10^

Despite this need, there remains a critical lack of symptom and functioning profiles to guide preoperative counseling for patients undergoing UGI surgery. No standardized benchmarks currently exist to define what “recovery” means from the patient’s perspective.^11^ The duration of symptom recovery, how it differs by the type of surgery performed, and which patient- and treatment-specific factors influence recovery are poorly understood.

To address this critical knowledge gap, we developed and validated the MD Anderson Symptom Inventory Survey for Patients with Upper Gastrointestinal Surgery (MDASI-UGI-Surg)^12^ using a rigorous, multiphase process aligned with U.S. Food and Drug Administration guidance, incorporating patient interviews, clinical expertise, and psychometric validation.^13,14^ As part of this tool development project, we conducted a prospective longitudinal study for detecting sensitivity of the new tool using PROs for patients undergoing UGI cancer surgery. Our aims were to (1) characterize longitudinal symptom burden to support data-driven preoperative counseling, (2) define recovery from the patient’s perspective to establish benchmarks for future patient-centered research, and (3) identify factors associated with delayed symptom recovery following UGI surgery.

## Methods

### Study Design and Oversight

This prospective longitudinal PRO cohort study at a single tertiary cancer center was conducted under a comprehensive protocol approved by The University of Texas MD Anderson Cancer Center Institutional Review Board (#2021-0799). The primary objective was to validate and assess the sensitivity of the newly developed MDASI-UGI-Surg in patients undergoing UGI cancer surgery. The results of the validation process are reported in an alternate manuscript.^15^

### Patients

All patients at MD Anderson aged ≥18 years could speak and read English and were scheduled to undergo curative-intent resection for esophageal, gastric, or pancreatic malignancies from February 2024 to September 2024 were screened for inclusion. Patients were excluded if they had cognitive impairment preventing reliable survey completion, required English translation, or underwent multivisceral resections involving more than one organ. Eligible patients were enrolled after providing informed consent. Those who did not complete the preoperative baseline survey or whose operations were cancelled or aborted were excluded from the final analysis.

### MDASI-UGI-Surg Instrument

The validated MDASI-UGI-Surg instrument^15^ consisted of 28 items, each scored on a 0–10 numeric scale. These items are classified into three groups: (1) core symptoms (13 general symptoms common across the cancer population), (2) module symptoms (nine symptoms specific to UGI surgical patients), and (3) interference with daily functioning items (six items assessing the impact of symptoms on daily functioning, further divided into a physical interference subscale [working, general activity, and walking] and an affective interference subscale [relations with other people, enjoyment of life, and mood]) (Supplementary Table S1).

Three composite scores were defined: (1) the mean scores of the five symptoms—selected from both core and module domains—that had the highest scores across the entire cohort on postoperative day (POD) 3; (2) the mean scores of the three interference items with the highest scores on POD3; and (3) the mean scores of six UGI-specific symptoms: nausea, lack of appetite, vomiting, diarrhea, constipation, and feeling of stomach fullness.^16^

Recovery was defined as achieving a mean score of  ≤ 3 for both the top five symptoms and the top three interference items, consistent with the thresholds from National Comprehensive Cancer Network clinical practice guidelines for fatigue and pain.^17,18^ The clinical validity of this cutoff has also been demonstrated in our previous studies using the MDASI survey in other cancer surgery populations.^19–21^

### Data Collection

Study data were collected and managed using REDCap electronic data capture tools (Vanderbilt University, Nashville, TN, USA) hosted at MD Anderson.^22,23^ Participants received email invitations to complete the MDASI-UGI-Surg at eight time points: preoperatively, at POD3, 7, 14, and 21, and at postoperative months (POM) 1, 3, and 6. Survey links expired after 48 h for the preoperative and POD3–21 time points, and after 2 weeks for POM1–6. The preoperative survey included sociodemographic variables (e.g., age, sex, race, ethnicity, marital status, highest education level, and employment status). Eastern Cooperative Oncology Group Performance Status^24^ was assessed and recorded by the treating provider and obtained through chart review. To maximize response rates through POM1, participants received follow-up reminders via telephone, email, and in-person contact. Clinical data (including treatment- and disease-specific variables) were abstracted through manual review of the electronic medical record.

### Statistical Analysis

Categorical variables were summarized as frequency and percentage; continuous variables were presented as mean ± standard deviation. Composite scores were summarized with mean and 95% confidence interval (CI) at each time point and visualized as line plots stratified by surgical cohort. Cumulative recovery was estimated through POM3 and POM6; for presentation, cumulative recovery up to POM1 was displayed using Kaplan–Meier curves, and groups were compared using the log-rank test. A minimally invasive approach was defined as thoracoscopic or robotic techniques for the thoracic portion of esophagectomy and as laparoscopic or robotic techniques for pancreatectomy or gastrectomy. Multivariable logistic regression was used to identify preoperative predictors of failure to recover by POM1. All statistical analyses were performed using R version 4.5.2 (R Foundation for Statistical Computing, Vienna, Austria). Two-sided *p-*values < 0.05 were considered statistically significant.

## Results

A total of 169 patients were enrolled in the study, 143 of whom were included in the final analysis. In total, 26 patients were excluded: seven because they did not complete the preoperative MDASI-UGI-Surg survey and 19 because their operations were cancelled or aborted. The demographic and clinical characteristics of the 143 evaluable patients are shown in Table 1. Their mean age was 61 years, 91 (64%) patients were male, and 108 (76%) were non-Hispanic white. The cohort included 42 (29%) patients who underwent esophagectomy, 27 (19%) gastrectomy, and 74 (52%) pancreatectomy.

**Table 1:** Patient characteristics

Characteristic		All cases (*n* = 143)	Esophagus (*n* = 42)	Stomach (*n* = 27)	Pancreas (*n* = 74)
Age, years		61.2 ± 13.3	59.9 ± 11.5	59.4 ± 14.9	62.5 ± 13.7
Sex					
	Male	91 (64)	35 (83)	13 (48)	43 (58)
	Female	52 (36)	7 (17)	14 (52)	31 (42)
Race and ethnicity					
	Non-Spanish, non-Hispanic white	108 (76)	38 (91)	14 (52)	56 (77)
	Non-Spanish, non-Hispanic Black	7 (5)	0	1 (4)	6 (8)
	Spanish or Hispanic	16 (11)	2 (5)	8 (30)	6 (8)
	Asian/Pacific Islander	10 (7)	2 (5)	3 (11)	5 (7)
	Other	1 (1)	0	1 (4)	1 (1)
Marital status					
	Married	115 (80)	34 (81)	20 (74)	61 (82)
	Single/never married	8 (6)	2 (5)	2 (7)	4 (5)
	Widowed	12 (8)	4 (10)	4 (15)	4 (5)
	Divorced	7 (5)	2 (5)	1 (4)	4 (5)
	Other/prefer not to say	1 (1)	0	0	1 (1)
Education					
	High school diploma	15 (11)	3 (7)	6 (22)	6 (8)
	Some college	20 (14)	7 (17)	4 (15)	9 (12)
	Associate’s degree	14 (10)	6 (14)	3 (11)	5 (7)
	Bachelor’s degree	51 (36)	18 (43)	7 (26)	26 (35)
	Master’s degree	25 (18)	6 (14)	6 (22)	13 (18)
	Professional/Doctorate degree	13 (9)	2 (5)	0	11 (15)
	Prefer not to say	5 (4)	0	1 (4)	4 (5)
Employment					
	Full-time employed	66 (46)	23 (55)	16 (60)	27 (37)
	Part-time employed	4 (3)	0	0	4 (5)
	Unemployed	3 (2)	1 (2)	0	2 (3)
	Self-employed	7 (5)	1 (2)	3 (11)	3 (4)
	Homemaker	6 (4)	0	1 (4)	5 (7)
	Retired	56 (39)	17 (41)	7 (26)	32 (42)
	Other/prefer not to say	1 (1)	0	0	1 (1)
ECOG PS (preoperative)					
	0	109 (76)	33 (79)	20 (74)	56 (76)
	1	31 (22)	8 (19)	7 (26)	16 (22)
	2	3 (2)	1 (2)	0	2 (3)
Type of surgery					
	Ivor Lewis esophagectomy	39 (27)	39 (93)	0	0
	Three-field esophagectomy with gastric conduit	3 (2)	3 (7)	0	0
	Total gastrectomy	8 (6)	0	8 (30)	0
	Distal gastrectomy	13 (9)	0	13 (48)	0
	Partial gastrectomy/wedge resection	6 (4)	0	6 (22)	0
	Pancreaticoduodenectomy	44 (31)	0	0	44 (59)
	Distal pancreatectomy	27 (19)	0	0	27 (37)
	Total pancreatectomy	2 (1)	0	0	2 (3)
	Central pancreatectomy	1 (1)	0	0	1 (1)
Thoracic approach					
	Open	30 (21)	30 (71)	0	0
	Thoracoscopic	1 (1)	1 (2)	0	0
	Robotic	11 (8)	11 (26)	0	0
Abdominal approach					
	Open	95 (66)	25 (60)	15 (56)	55 (74)
	Laparoscopic	2 (1)	1 (2)	0	0
	Robotic	46 (32)	16 (38)	12 (44)	19 (26)
Histology					
	Adenocarcinoma	101 (71)	36 (86)	19 (70)	46 (62)
	Carcinoid/neuroendocrine	18 (13)	0	2 (7)	16 (22)
	Cystic disease	10 (7)	0	0	10 (14)
	GIST	6 (4)	0	6 (22)	0
	Squamous cell carcinoma	4 (3)	4 (10)	0	0
	Metastatic lesion(s) from other organs	2 (1)	0	0	2 (3)
	Adenosquamous carcinoma	1 (1)	1 (2)	0	0
	Benign stricture	1 (1)	1 (2)	0	0
Neoadjuvant treatment					
	None (upfront)	45 (32)	2 (5)	12 (44)	31 (42)
	Chemotherapy only	41 (29)	11 (26)	9 (33)	21 (28)
	Chemoradiotherapy	57 (40)	29 (69)	6 (22)	21 (30)
Adjuvant chemotherapy		57 (40)	20 (48)	6 (22)	31 (42)
Tumor size, cm^a^		3.8 ± 2.6	4.5 ± 2.9	3.2 ± 3.0	3.5 ± 2.0
pT^a^					
	≤1	39 (30)	17 (42)	10 (37)	12 (19)
	2	46 (35)	8 (20)	7 (26)	31 (48)
	3	38 (29)	13 (32)	6 (22)	19 (30)
	4	6 (5)	2 (5)	3 (11)	1 (2)
	Unknown or not applicable	3 (2)	1 (2)	1 (2)	1 (2)
pN^a^					
	0	69 (52)	23 (56)	17 (63)	29 (45)
	1	39 (30)	11 (27)	5 (19)	23 (36)
	2/3	22 (17)	7 (17)	3 (11)	12 (19)
	Unknown or not applicable	2 (2)	0	2 (7)	0
Margin status^a^					
	R0	126 (96)	40 (98)	26 (96)	60 (94)
	R1	5 (4)	1 (2)	1 (2)	3 (5)
	R2	1 (1)	0	0	1 (2)
Complications by Clavien–Dindo grade					
	<II	106 (74)	32 (76)	25 (93)	49 (66)
	IIIa	25 (18)	4 (10)	2 (7)	19 (26)
	IIIb	7 (5)	4 (10)	0	3 (4)
	IV/V	5 (4)	2 (5)	0	3 (4)
Reintervention					
	None	109 (76)	33 (79)	25 (93)	51 (69)
	Endoscopic	9 (6)	5 (12)	0	4 (5)
	Radiologic	23 (16)	1 (2)	2 (7)	20 (27)
	Surgical	7 (5)	4 (10)	0	3 (4)
Length of hospital stay, days		7.9 ± 4.9	9.2 ± 3.6	5.6 ± 3.6	8.0 ± 5.5
Readmission within 90 days		49 (34)	14 (33)	6 (22)	29 (39)

Data are presented as n (%) or mean ± standard deviation unless otherwise indicated

ECOG PS, Eastern Cooperative Oncology Group Performance Status; GIST, gastrointestinal stromal tumor

^a^n = 132, excluding cystic disease and benign stricture

The most common operations included were pancreatoduodenectomy (*n* = 44; 31%), Ivor Lewis esophagectomy (*n* = 39; 27%), distal pancreatectomy (*n* = 27; 19%), and distal gastrectomy (*n* = 13; 9%). A minimally invasive approach was used in 43 (30%) of the patients. In total, 41 (29%) patients received neoadjuvant chemotherapy alone, 56 (39%) received neoadjuvant chemoradiotherapy, and 57 (40%) received adjuvant chemotherapy; 37 (26%) patients experienced Clavien–Dindo grade III or higher complications,^25^ and 49 (34%) were readmitted within 90 days after surgery.

**Fig. 1:**
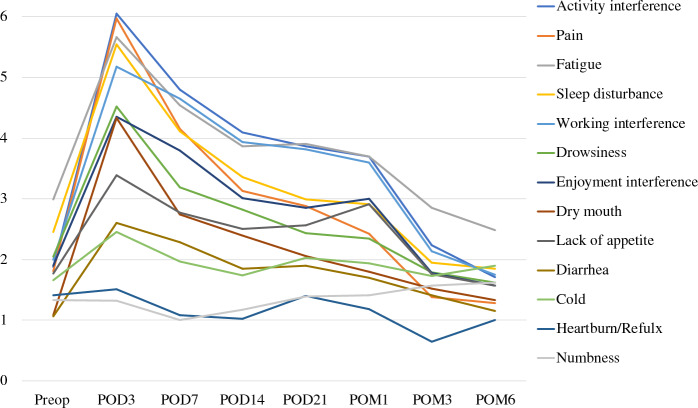
Mean score trajectories of selected MDASI-UGI-Surg symptoms and interference items over time, before and after surgery. Symptoms/items are ordered by their mean scores on postoperative day (POD) 3. Higher scores indicate greater severity. POM, postoperative month

All 143 patients completed at least two postoperative MDASI-UGI-Surg surveys. Response rates were high ( ≥ 92%) from the preoperative time point through POM1 but declined to 63–64% at POM3 and 6 (Supplementary Table S2). The severity and trajectories of the mean scores for selected individual symptoms and interference items are shown in Fig. 1. Mean score trajectories for all symptoms and interference items are shown in Supplementary Figure 1. Most of the scores peaked on POD3. The highest mean symptom scores on POD3 were pain (6.0), fatigue (5.7), sleep disturbance (5.6), drowsiness (4.5), and dry mouth (4.3), representing the top five symptoms. The top three interference items were general activity (6.1), working (5.2), and enjoyment of life (4.4).

Symptom recovery followed a consistent and structured pattern across the cohort, characterized by three distinct phases. In the acute phase (POD3–14), symptoms improved rapidly. This was followed by a plateau phase (POD14–POM1), during which symptom burden remained relatively stable with minimal further improvement. Among the symptom scores at POM1, fatigue (3.7), activity interference (3.7), and work interference (3.6) were higher than the other symptoms. In the persistent recovery phase, which came after POM1, most symptoms began to exhibit further improvement, continuing through POM3 and 6. Notably, fatigue remained more persistent than other symptoms, with mean scores of 2.9 at POM3 and 2.5 at PO6, both of which were higher than other symptoms at those time points. Chemotherapy-related symptoms, such as feeling cold and numbness, became more prominent during this later phase (Fig. 1).

Table 2 presents the mean scores for individual symptoms, interference items, and composite scores at POD3 and POM1 stratified by organ group. Overall, symptom severity was similar across esophagectomy, gastrectomy, and pancreatectomy groups. The top five symptoms and top three interference items were consistent across groups and time points. Some organ-specific differences were noted: the esophagectomy patients reported higher mean scores for shortness of breath, dry mouth, and difficulty swallowing, whereas the pancreatectomy patients reported higher scores for lack of appetite and diarrhea. However, these differences were small and clinically minor.

**Table 2:** Mean scores for each symptom/item in the MDASI-UGI-Surg on postoperative day (POD) 3 and postoperative month (POM) 1

Category	Question number	Item	POD 3				POM 1			
			All cases	Esophagus	Stomach	Pancreas	All cases	Esophagus	Stomach	Pancreas
Core symptoms	1	Pain	**6.0**	**6.7**	**5.4**	**5.7**	**2.4**	**3.2**	1.5	**2.4**
	2	Fatigue	**5.7**	**6.2**	**5.6**	**5.3**	**3.7**	**3.7**	**3.1**	**3.9**
	3	Nausea	2.0	1.7	2.6	1.9	1.2	1.2	0.9	1.4
	4	Sleep disturbance	**5.6**	**5.8**	**5.9**	**5.3**	**2.9**	**3.2**	**2.4**	**3.0**
	5	Distress	3.2	3.6	3.0	3.0	1.8	1.9	1.5	1.8
	6	Shortness of breath	2.8	3.6	2.4	2.4	1.3	1.6	1.0	1.3
	7	Problem with remembering things	2.1	2.0	2.4	2.0	1.2	0.9	1.6	1.2
	8	Lack of appetite	3.4	2.8	3.2	3.8	**2.9**	**2.6**	**2.5**	**3.3**
	9	Drowsiness	**4.5**	**4.3**	**4.6**	**4.6**	2.3	2.5	**2.3**	2.3
	10	Dry mouth	**4.3**	**5.3**	3.4	**4.1**	1.8	2.3	1.6	1.6
	11	Sadness	2.1	2.5	2.0	1.9	1.5	1.8	1.0	1.5
	12	Vomiting	0.5	0.8	0.4	0.3	0.5	0.7	0.3	0.6
	13	Numbness or tingling	1.3	1.0	1.4	1.5	1.4	1.3	1.1	1.6
Module symptoms	14	Difficulty swallowing	2.0	3.3	1.2	1.6	0.9	1.4	0.5	0.7
	15	Heartburn or reflux	1.5	1.2	1.1	1.9	1.2	1.5	0.8	1.2
	16	Diarrhea	2.6	1.4	2.9	3.2	1.7	1.6	1.4	1.8
	17	Constipation	2.3	2.4	3.8	1.7	1.3	1.7	1.2	1.1
	18	Feeling cold	2.5	2.0	3.3	2.4	1.9	1.6	1.7	2.2
	19	Flushing or sweating	1.9	2.0	1.8	1.8	0.9	0.8	0.9	0.9
	20	Stomach feeling full	3.2	1.9	3.8	3.8	**2.9**	**2.9**	**2.3**	**3.0**
	21	Malaise	3.8	3.8	**4.1**	3.7	2.2	2.4	1.6	2.3
	22	Dizziness	1.7	1.4	2.1	1.7	1.1	1.0	1.0	1.2
Interference items	23	General activity	*6.1*	*6.1*	*5.8*	*6.1*	*3.7*	*3.8*	*3.1*	*3.9*
	24	Mood	3.8	3.6	3.5	4.0	2.7	2.7	2.0	2.9
	25	Working	*5.2*	*5.2*	*5.5*	*5.1*	*3.6*	*3.6*	*3.1*	*3.8*
	26	Relations with other people	3.0	2.7	2.9	3.1	1.8	1.7	1.6	1.8
	27	Walking	4.0	3.8	3.7	4.3	2.4	2.3	1.7	2.7
	28	Enjoyment of life	*4.4*	*4.3*	*4.2*	*4.4*	*3.0*	*3.3*	*2.3*	*3.1*
Composite score	Top five symptoms^a^		5.2	5.7	5.0	5.0	2.6	3.0	2.2	2.6
	Top three interference items^b^		5.2	5.2	5.1	5.2	3.4	3.5	2.8	3.6
	UGI-specific symptoms^c^		2.3	1.8	2.8	2.5	1.8	1.8	1.4	1.9

Bold indicates the worst five symptoms and three interference items for each group and timing. Italic indicates the worst three interference items for each group and timing

UGI, upper gastrointestinal

^a^Pain, fatigue, sleep disturbance, drowsiness, and dry mouth

^b^Activity, working, and enjoyment of life

^c^Nausea, lack of appetite, vomiting, diarrhea, constipation, and stomach full

**Fig. 2:**
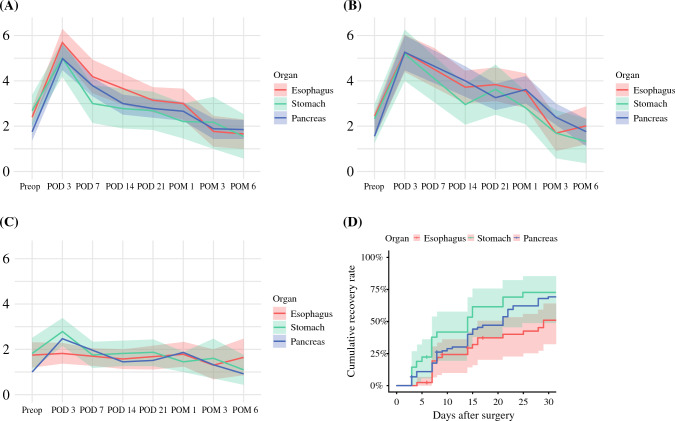
Trends in composite scores and their 95% confidence intervals (CIs), stratified by organ (red: esophagus, green: stomach, blue: pancreas) over time. **A** Mean composite scores for the top five symptoms among core and module symptoms with the highest mean scores on postoperative day (POD) 3 (pain, fatigue, sleep disturbance, drowsiness, and dry mouth). **B** Mean composite scores for the top three interference items with the highest mean scores on POD 3 (general activity, working, and enjoyment of life). **C** Mean composite scores for the six upper gastrointestinal (UGI)-specific symptoms (nausea, lack of appetite, vomiting, diarrhea, constipation, and stomach full). **D** Kaplan–Meier curves with 95% CIs illustrating cumulative recovery for the three organs up to postoperative month (POM) 1. Recovery was defined as achieving both mean scores of  ≤ 3 for the top five symptoms and the top three interference items

Cumulative symptom recovery rates—defined as achieving a mean score of ≤3 for both the top five symptoms and top three interference items—were 64.8% at POM1, 78.9% at POM3, and 90.8% at POM6. When stratified by organ groups, recovery rates at POM1, 3, and 6 were 52.2, 77.7, and 94.4% for esophagectomy; 70.4, 74.1, and 90.1% for gastrectomy; and 69.6, 82.1, and 90.1% for pancreatectomy, respectively. These trajectories are shown in Fig. 2a-c, which displays trends in the mean composite scores and their 95% CIs by organ group. Among the three organ groups, patients undergoing esophagectomy had modestly higher symptom burden on the top five symptom composite from POD3 through POM1 (Fig. 2a). In contrast, the top three interference composite and the UGI-specific symptom composite scores followed similar trajectories across the three groups (Fig. 2b and c). Kaplan–Meier analysis confirmed a significant difference in cumulative recovery rates by organ group (log-rank *p* = 0.049; Fig. 2d).

In exploratory analyses, we examined recovery trends across the five most frequently performed procedures: total gastrectomy, distal gastrectomy, pancreatoduodenectomy, distal pancreatectomy, and Ivor Lewis esophagectomy. We observed notable variation in symptom trajectories, particularly between total and distal gastrectomy (Supplementary Figure S2a). When stratified by neoadjuvant treatment, consisting of no treatment, neoadjuvant chemotherapy only, and neoadjuvant chemoradiotherapy, the symptom trends were generally similar (Supplementary Figure S2b). Trajectories were also comparable when stratified by receipt of adjuvant therapy (Supplementary Figure S2c). In contrast, patients who experienced postoperative complications exhibited markedly worse symptom trajectories (Supplementary Figure S2d). Although subject to selection bias and confounding by operation type, use of a minimally invasive approach appeared to attenuate symptom burden during the acute and plateau phases of recovery (Supplementary Figure S3a–c).

In univariable analysis, postoperative complications and 90 day readmissions were both strongly associated with failure to achieve symptom recovery at POM1, each with an odds ratio of 2.5 (*p* < 0.05). In the multivariable model limited to preoperative factors, the type of surgery remained the only independent predictor of failure to recover at POM1 (*p* = 0.039), whereas surgical approach (open vs. minimally invasive) was not statistically significant (odds ratio 2.43; 95% CI 0.99–6.50; *p* = 0.062), though the point estimate suggests a fairly strong effect.

## Discussion

This prospective study provides one of the most detailed and comprehensive evaluations of postoperative symptom recovery in patients undergoing UGI cancer surgery. With exceptionally high response rates, we demonstrated the excellent sensitivity of the newly developed, procedure-specific MDASI-UGI-Surg instrument in capturing longitudinal PRO profiles across multiple UGI organs and procedures. These data enabled us to characterize key phases of symptom evolution, establish patient-centered recovery benchmarks, and identify that recovery greatly varied by the type of surgery and incidence of complications but not by the use of neoadjuvant therapy.

Notably, we identified three distinct phases of recovery: a rapid improvement during the acute phase (POD3–14), a plateau in symptom burden during the intermediate phase (POD14–POM1), and a persistent recovery phase extending through POM3 and 6. Although these trajectories align with clinical experience, we are the first to formally quantify these transitions using standardized PRO metrics. Furthermore, we established a definition for postoperative symptom recovery after UGI cancer surgery, providing a benchmark for future studies. Although this definition has certain limitations—such as the mean score of selected items potentially failing to reflect a few clinically relevant symptoms and the cumulative recovery rate possibly overlooking symptom relapse—and may not fully capture patients’ perceptions of complete recovery, it nevertheless provides a landmark for the assessment of symptom recovery. These findings underscore the importance of incorporating longitudinal PRO assessments into survivorship planning and follow-up care.

Although the study was not powered to detect definitive differences by surgical procedure or approach, we observed notable differences in recovery patterns. A minimally invasive approach appeared to mitigate symptom burden in the acute and plateau phases. However, it was not independently associated with recovery at POM1 in the multivariable analysis after adjustment by surgical type. In contrast, postoperative complications and readmissions were strongly associated with symptom recovery failure, reinforcing the impact of perioperative morbidity on PROs. These insights may guide preoperative counseling, risk stratification, and individualized recovery planning (Table 3).

**Table 3:** Results of univariable and multivariable analysis evaluating risk factors for symptom recovery failure at postoperative month (POM) 1

Variable	Univariable analysis				Multivariable analysis			
	Odds ratio	95% CI		*P*-value	Odds ratio	95% CI		*P*-value
Age <65 (vs. ≥65) years	1.70	0.83	3.56	0.148				
Female sex (vs. male)	1.03	0.49	2.13	0.945				
Non-Spanish, non-Hispanic white (vs. others)	1.57	0.70	3.74	0.29				
Married status (vs. others)	2.03	0.79	5.91	0.161				
Any degrees or higher education (vs. some college or less)	1.74	0.78	4.14	0.188				
Unemployed (vs. employed)	0.99	0.49	2.00	0.979				
Preoperative ECOG PS ≥2 (vs. 0–1)	3.83	0.36	83.62	0.278				
Type of surgery (vs. distal/central pancreatectomy)				0.009				0.039
Esophagectomy	5.70	1.80	22.18		5.31	1.65	20.94	
Total gastrectomy	6.00	1.06	37.43		4.88	0.84	31.07	
Distal/partial gastrectomy	1.20	0.21	6.19		1.42	0.24	7.56	
Pancreatoduodenectomy/total pancreatectomy	4.15	1.33	15.96		3.45	1.07	13.46	
Open approach^a^ (vs. minimally invasive approach)	3.09	1.34	7.82	0.011	2.43	0.99	6.50	0.062
Histology of adenocarcinoma (vs. others)	1.84	0.82	4.36	0.149				
Neoadjuvant treatment (vs. none)	1.23	0.58	2.70	0.587				
*Postoperative factors: not included in multivariable analysis*								
Adjuvant chemotherapy (vs. none)	0.88	0.43	1.79	0.724				
Grade ≥III postoperative complications (vs. grade ≤II)	2.50	1.12	5.63	0.025				
Readmission within 90 days	2.49	1.20	5.24	0.015				

Recovery was defined as achieving both a mean score of ≤3 for the top five symptoms and the top three interference items within POM 1

CI, confidence interval; ECOG PS, Eastern Cooperative Oncology Group Performance Status

^a^Esophagus is based on the thoracic approach

Our findings offer evidence that provides practical guidance for clinicians and patients to better understand expected symptom management period and set appropriate recovery expectations after UGI surgery, including return to normal activities.^26^ Specifically, this study provides data-driven reference points that can be used to explain common postoperative symptoms, reassure patients experiencing prolonged fatigue, and inform shared decision-making regarding additional treatments, such as adjuvant therapy and treatment of recurrence.^27^ Additionally, real-time symptom monitoring using this tool may facilitate early detection of complications and support postoperative self-management strategies.^28^

A major contribution of this study is the use of the novel, reliable, and UGI-specific MDASI-UGI-Surg instrument. Widely used general PRO instruments, such as the 36-Item Short Form Health Survey (SF-36),^29^ The European Organisation for Research and Treatment of Cancer Core Quality of Life Questionnaire (QLQ-C30),^30^ and the EQ-5D,^31^ lack sensitivity regarding symptom recovery specific to UGI surgery and often require the addition of disease-specific modules (e.g., QLQ-STO22 or OES18), resulting in lengthy surveys exceeding 50 items.^32,33^ This leads to survey fatigue and poor compliance.^34^ In contrast, the MDASI-UGI-Surg consists of 28 questions, contributing to the high response rates observed in this study, which were further supported by active engagement from our research collaborators.

Our next goal is to further enhance the tool’s clinical applicability by reducing its length while preserving its validity and sensitivity. We are currently conducting further cluster analyses to identify symptom domains based on severity and dynamics with the goal of creating the MDASI-UGI-Short, a version containing fewer than 15 items. This abbreviated tool will be suitable for integration into routine clinical workflows in both inpatient and outpatient settings, facilitating near-universal completion and promoting patient-centered surgical care for patients with UGI cancers. Moreover, additional validation is required to confirm that the recovery definition employed in this study corresponds with patients’ perceptions of recovery, thereby ensuring its relevance to meaningful, patient-centered outcomes.

Despite the considerable strengths of this study—including its prospective design, detailed longitudinal data, and high PRO response rates in a large patient cohort—several limitations should be acknowledged. First, the study was conducted at a single tertiary referral center with an exclusively English-speaking population, which may limit the generalizability of the findings. To address this, we plan to translate and culturally adapt the MDASI-UGI-Surg for use in other languages and international settings. Second, although our organ-specific analyses provided useful insights, the sample sizes within each surgical subgroup limited our ability to fully evaluate differences by the type of and approach to surgery. Third, we did not evaluate the impact of perioperative pain management strategies (e.g., epidural, regional blocks, opioid use) or other intra- and perioperative factors (e.g., transfusion requirement, operative time, length of stay) on recovery trajectories.

Ongoing efforts to integrate the MDASI-UGI-Surg—and the forthcoming MDASI-UGI-Short—into routine electronic health record systems will facilitate continuous data collection and support more granular future analyses. Planned analyses using larger, real-world datasets will specifically examine the impact of different surgical approaches. Although our multivariable analysis did not identify a significant association between surgical approach and recovery at POM1 in the overall cohort, the effect of minimally invasive approach may vary by procedure type, with potential quality-of-life benefits more apparent during the early recovery phase in certain subgroups. Continued data accumulation and procedure-specific analyses are needed to clarify the role and potential advantages of minimally invasive approach in UGI cancer operations.

## Conclusions

Our study provides essential patient-centered data on the trajectory of symptom recovery after UGI cancer surgery. These findings fill a critical knowledge gap in perioperative care, offering data-driven benchmarks to support patient counseling, enhance recovery pathways, and improve shared decision-making. The MDASI-UGI-Surg is a valuable tool for integrating the patient’s voice into perioperative care, quality improvement initiatives, and future clinical trials.

## Supplementary Information

Below is the link to the electronic supplementary material.

Supplementary file1 Fig. S1 Mean score trajectories of all MDASI-UGI-Surg symptoms and interference items over time, before and after surgery. Symptoms/items are ordered by their mean scores on POD 3. Higher scores indicate greater severity: Fig. S2Trends in the mean composite scores for the top five symptoms and their 95% CIs over time. Shown are the trends according to (a) type of surgery (red: Ivor Lewis esophagectomy; gold: total gastrectomy; green: distal gastrectomy; blue: pancreatoduodenectomy; pink: distal pancreatectomy), (b) neoadjuvant treatment (red: none (upfront surgery); green: chemotherapy only; blue: chemoradiotherapy), (c) adjuvant chemotherapy (red: no; light blue: yes), and (d) postoperative complications (Clavien-Dindo grade: red, ≤II; light blue, ≥III): Fig. S3Trends in the mean composite scores for the top five symptoms and their 95% CIs over time according to organ group. (a) Esophagus (based on thoracic approach); (b) stomach; (c) pancreas. All minimally invasive approaches to gastric and pancreatic surgery were performed robotically. Red: open approach; light blue: minimally invasive approach

Supplementary file2 (DOCX 37 KB)
