# Supplementary figures and images for: Patient-Reported Symptom Recovery After Upper Gastrointestinal Cancer Surgery: A Prospective Study Using the MDASI-UGI-Surg
Source: Ann Surg Oncol. 2026 Feb 19;33(6):5703–13. doi: 10.1245/s10434-026-19282-0 (PMC13179234; doi:10.1245/s10434-026-19282-0)

Suppl. Fig. 1

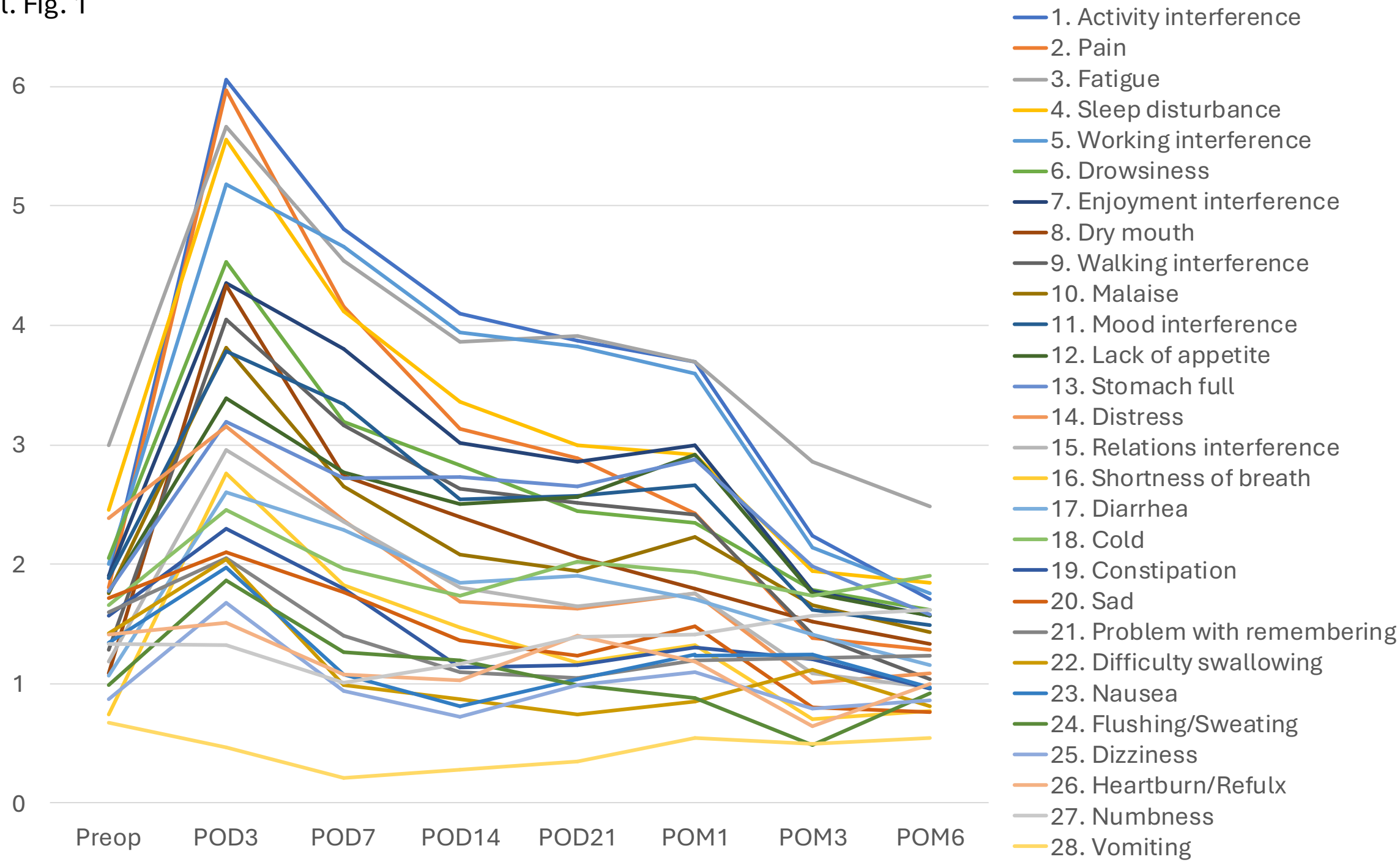

Suppl. Fig. S2a

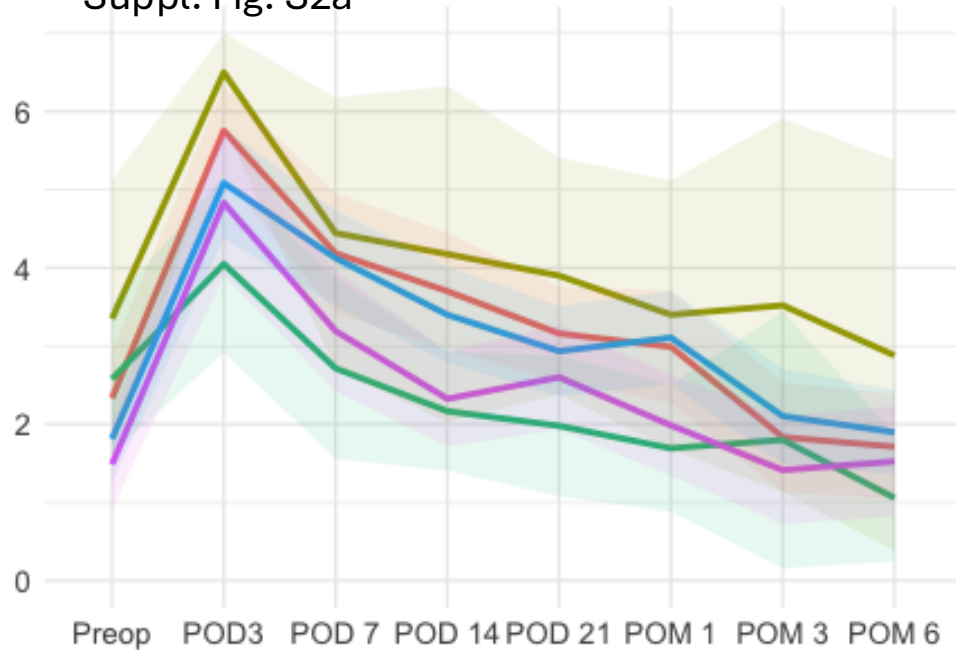

Suppl. Fig. S2b

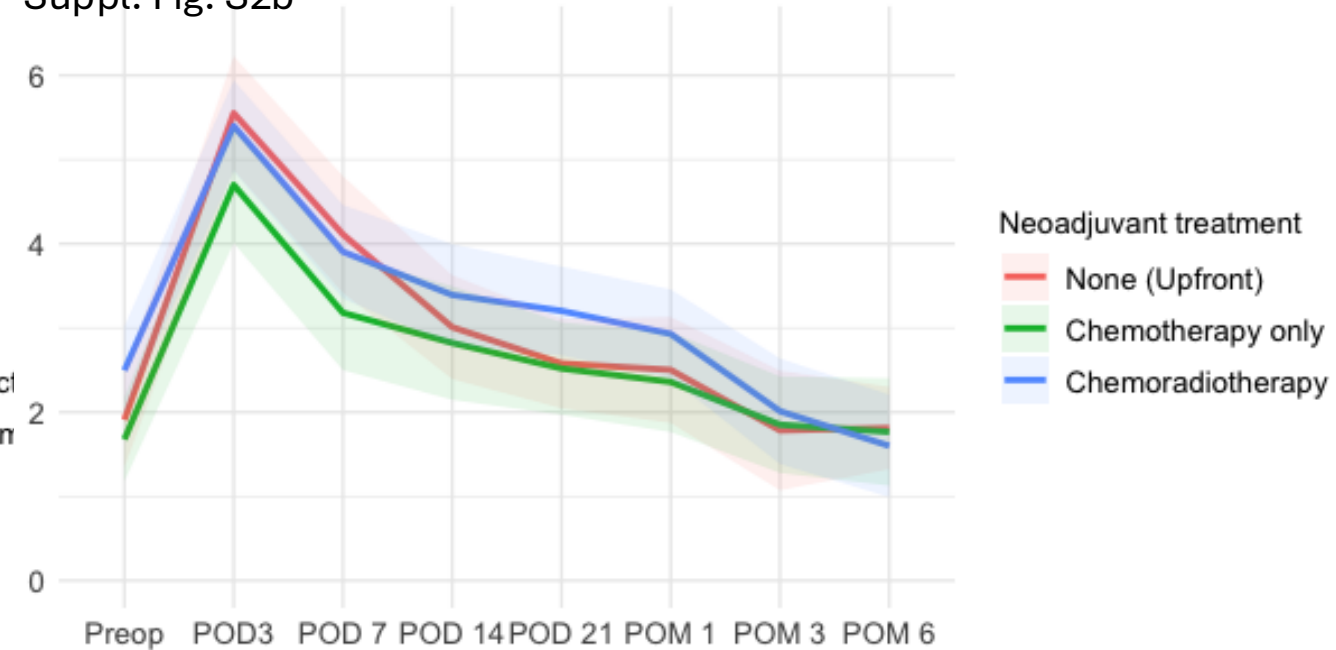

Suppl. Fig. S2c

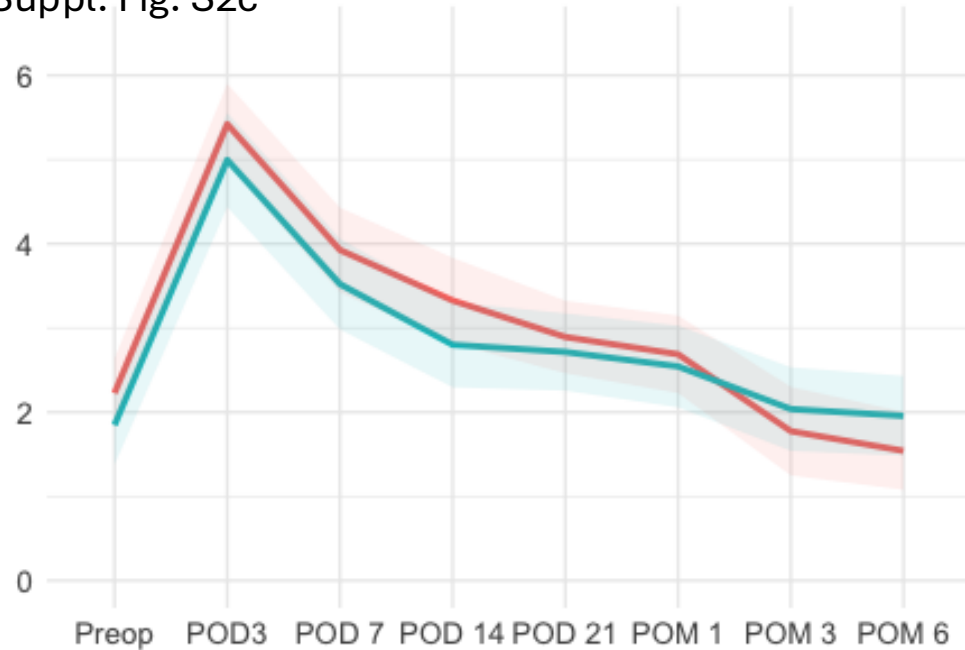

Suppl. Fig. S2d

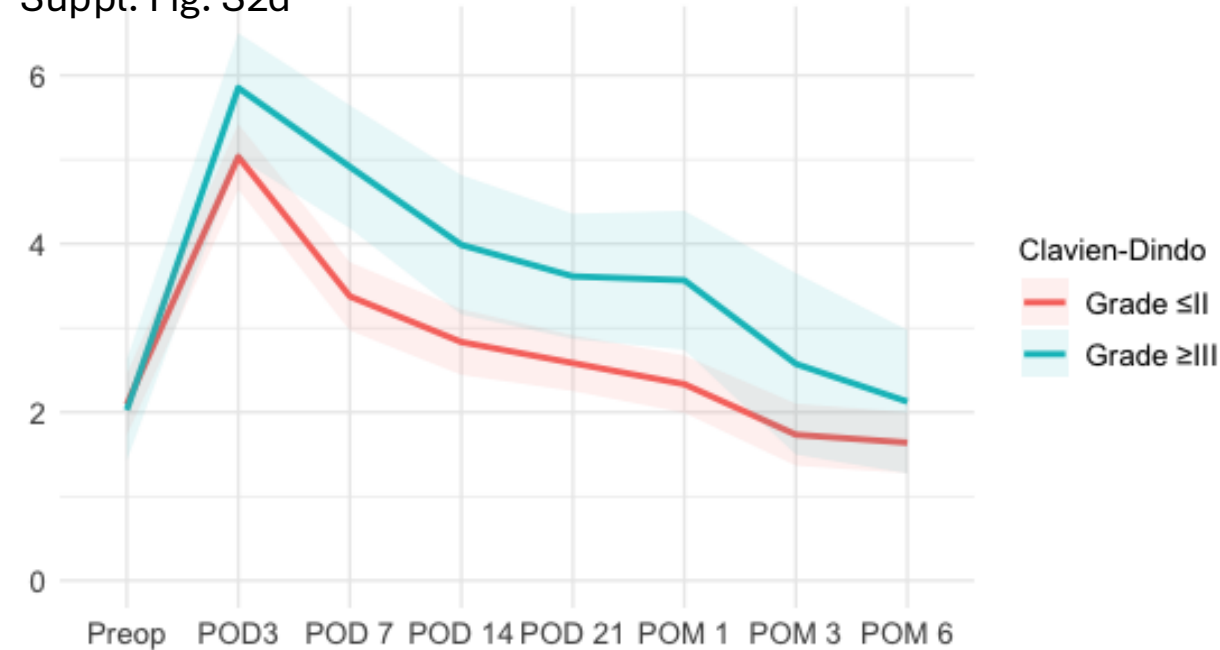

Suppl. Fig. S3a

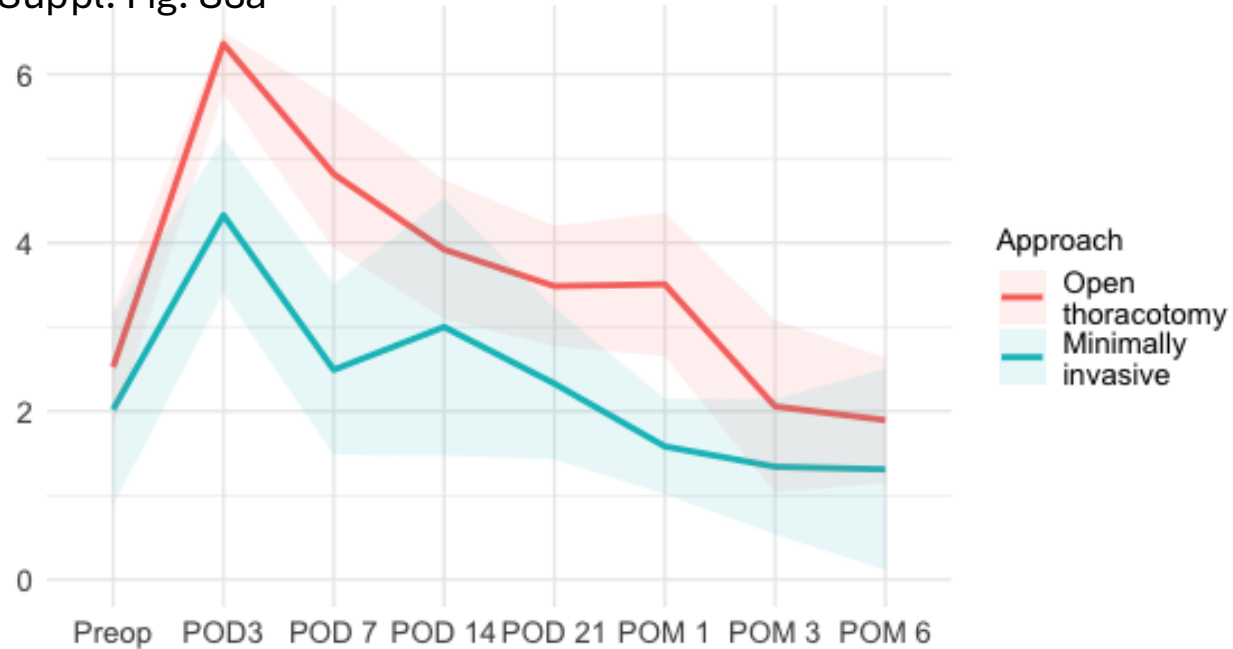

Suppl. Fig. S3b

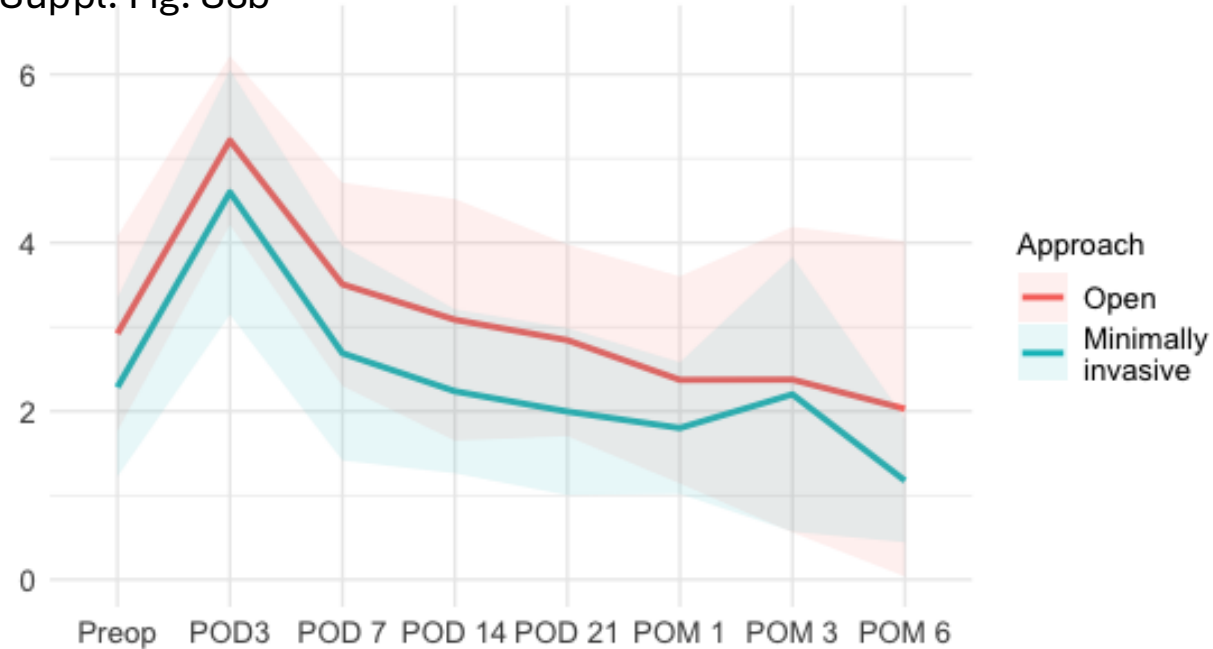

Suppl. Fig. S3c

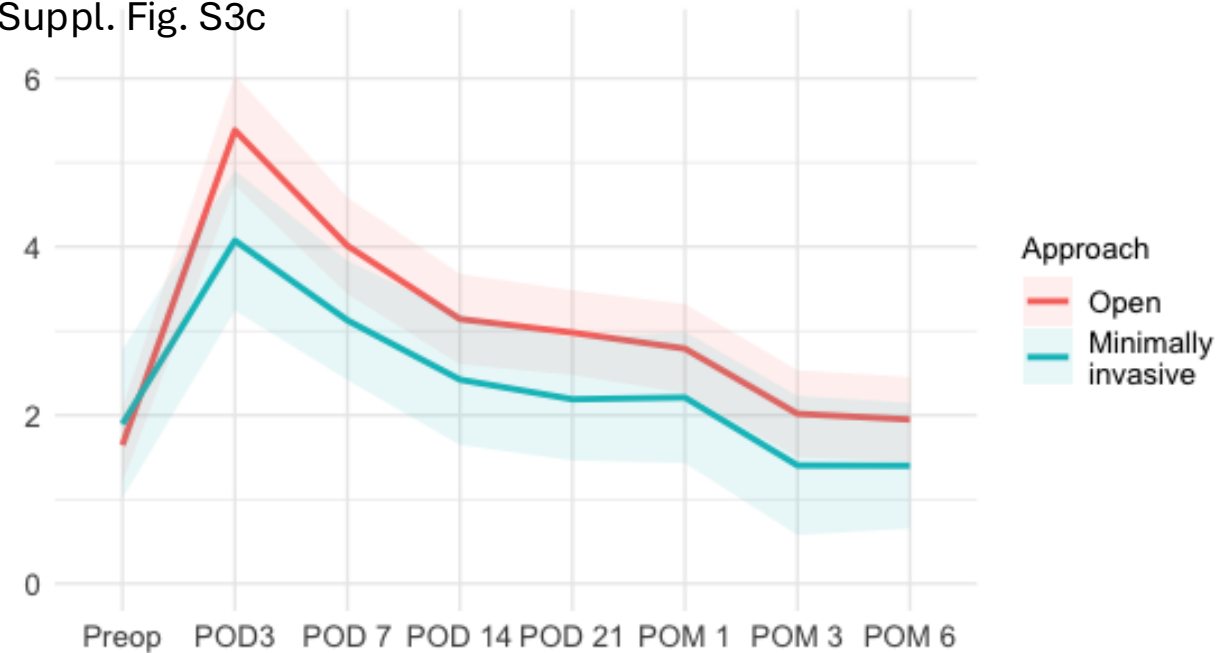

Supplement: Supplementary file 1 — Supplementary file1 Fig. S1 Mean score trajectories of all MDASI-UGI-Surg symptoms and interference items over time, before and after surgery. Symptoms/items are ordered by their mean scores on POD 3. Higher scores indicate greater severity: Fig. S2Trends in the mean composite scores for the top five symptoms and their 95% CIs over time. Shown are the trends according to (a) type of surgery (red: Ivor Lewis esophagectomy; gold: total gastrectomy; green: distal gastrectomy; blue: pancreatoduodenectomy; pink: distal pancreatectomy), (b) neoadjuvant treatment (red: none (upfront surgery); green: chemotherapy only; blue: chemoradiotherapy), (c) adjuvant chemotherapy (red: no; light blue: yes), and (d) postoperative complications (Clavien-Dindo grade: red, ≤II; light blue, ≥III): Fig. S3Trends in the mean composite scores for the top five symptoms and their 95% CIs over time according to organ group. (a) Esophagus (based on thoracic approach); (b) stomach; (c) pancreas. All minimally invasive approaches to gastric and pancreatic surgery were performed robotically. Red: open approach; light blue: minimally invasive approach [file 10434_2026_19282_MOESM1_ESM.pdf]
