# Supplementary material for: Patient-Reported Symptom Recovery After Upper Gastrointestinal Cancer Surgery: A Prospective Study Using the MDASI-UGI-Surg
Source: Ann Surg Oncol. 2026 Feb 19;33(6):5703–13. doi: 10.1245/s10434-026-19282-0 (PMC13179234; doi:10.1245/s10434-026-19282-0)
Supplement: Supplementary file 2 — Supplementary file2 (DOCX 37 KB) [file 10434_2026_19282_MOESM2_ESM.docx]

**Supplementary Table S1. MDASI-UGI-Surg**

| Core symptoms | |
| --- | --- |
|  | Pain |
|  | Fatigue |
|  | Nausea |
|  | Sleep disturbance |
|  | Distress |
|  | Shortness of breath |
|  | Problem with remembering things |
|  | Lack of appetite |
|  | Drowsiness |
|  | Dry mouth |
|  | Sadness |
|  | Vomiting |
|  | Numbness or tingling |
| Module symptoms | |
|  | Difficulty swallowing |
|  | Heartburn or reflux |
|  | Diarrhea |
|  | Constipation |
|  | Feeling cold |
|  | Flushing or sweating |
|  | Stomach feeling full |
|  | Malaise |
|  | Dizziness |
| Interference with daily functioning items | |
|  | General activity |
|  | Mood |
|  | Working (including work around the house) |
|  | Relations with other people |
|  | Walking |
|  | Enjoyment of life |
| These questions are being asked in simple and consistent language, such as “How severe are your symptoms (scale 0-10) when they are at their worst?” and “How much have the following items interfered with your life (scale 0-10)?”. Patients are asked to recall the severity of their symptoms over the past 24 hours. | |

**Supplementary Table S2. MDASI-UGI-Surg survey response rates**

|  | *n* | Preop | POD 3 | POD 7 | POD 14 | POD 21 | POM 1 | POM 3 | POM 6 |
| --- | --- | --- | --- | --- | --- | --- | --- | --- | --- |
| All cases | 143 | 100% | 96% | 97% | 92% | 92% | 96% | 63% | 64% |
| Esophagus | 42 | 100% | 100% | 95% | 93% | 88% | 90% | 57% | 55% |
| Stomach | 27 | 100% | 93% | 100% | 100% | 96% | 100% | 56% | 52% |
| Pancreas | 74 | 100% | 95% | 97% | 89% | 93% | 97% | 69% | 73% |
